# Knowledge, Attitude, and Practices Toward Tuberculosis Among Hospital Outpatients in Kabul, Afghanistan

**DOI:** 10.3389/fpubh.2022.933005

**Published:** 2022-07-11

**Authors:** Mohammad Yasir Essar, Khalid Jan Rezayee, Shoaib Ahmad, Manar Ahmed Kamal, Reshaduddin Nasery, Tamim Jan Danishmand, Michael Head, Arash Nemat

**Affiliations:** ^1^Afghanistan National Charity Organization for Special Diseases, Kabul, Afghanistan; ^2^Clinical Informatics Research Unit, Faculty of Medicine, University of Southampton, Southampton, United Kingdom; ^3^Department of Medical Laboratory Technology, Kabul University of Medical Sciences, Kabul, Afghanistan; ^4^District Head Quarter Teaching Hospital, Faisalabad, Pakistan; ^5^Faculty of Medicine, Benha University, Benha, Egypt; ^6^Surveillance Officer at Swedish Committee for Afghanistan, Parwan Management Office, Parwan, Afghanistan; ^7^Surveillance Department, Ministry of Public Health, Kabul, Afghanistan; ^8^Department of Microbiology, Kabul University of Medical Sciences, Kabul, Afghanistan

**Keywords:** tuberculosis, healthcare system, Afghanistan, COVID-19, crisis

## Abstract

**Background:**

Tuberculosis (TB) is a high-burden respiratory infectious disease. There was a sharp decline in the number of confirmed TB cases during the pandemic; this is likely to be influenced by the COVID-19 pandemic response, with under-reporting due to resource diversion. There are typically 13,000 tuberculosis-associated deaths in Afghanistan annually, with significant problems posed by drug-resistant TB.

**Method:**

A cross-sectional descriptive study was conducted in Afghanistan on Kabul residents who visited the adult outpatient departments of public hospitals for any health-related reason from 1st January to 20th March 2022. The study scored their knowledge, attitude, and practices (KAP) toward tuberculosis. The sample size was calculated using Epi-Info, and the minimum sample size was 385. The sampling method is chosen the non-probability convenient sampling for data gathering. Data were analyzed using SPSS version 28, and we used the Mann-Whitney test, Chi-square or fisher extract test, spearman correlations, and binary logistic regression model.

**Results:**

Of 829 participants, 450 (54.3%) were males and 379 (45.7) females. The median age was 28 years, and 63.3% were married. Most participants were unemployed (75.5%), but 54% had a monthly income >3,000 Afghanis, indicating the reliance on family. By TB knowledge score, 727 (87.7%) participants had good knowledge, and 800 (96.5%) participants had a positive attitude toward treatment and control. Only 2 participants reported poor practices regarding prevention. Regarding the binary logistic regression, young age, being a male, belonging to the “1,000–3,000” Afghani monthly income category, and having a positive attitude were significant predictors of good TB knowledge (*P*-value = 0.009, 0.000, 0.003, and 0.009), respectively. A positive attitude was expected to have good knowledge 6.035 times more than a negative attitude (95% CI: 1.572–23.167).

**Conclusion:**

The study findings highlighted that outpatients in Kabul had good knowledge, attitude, and practice toward TB. More studies are needed to highlight KAP in different Afghan populations, including in other parts of the country.

## Introduction

Tuberculosis (TB) is a bacterial infection responsible for ~1.5 m deaths in 2020 (1). Transmission is *via* respiratory droplets and aerosols from one person to another, including coughing, sneezing, fever, and headache (2). On average, one TB patient can infect 10–15 people (3). On-time screening and medications are essential for managing TB to reduce the likelihood of multidrug-resistant TB. Multidrug-resistant TB is a growing public health challenge that emerges if mismanagement in the treatment of TB occurs (4). With the emergence of the COVID-19 pandemic, there was a global disruption in many areas of healthcare and surveillance, including TB control programs; this is more visible in countries with the highest burden of TB- a sharp decline in confirmed TB cases was observed during 2020 and 2021, likely to be due to under-reporting (5).

The COVID-19 outbreak has undone decades of progress in the fight against TB, according to the Global Tuberculosis Report 2021. TB fatalities have surged for the first time in more than 10 years. In addition, fewer patients than in 2019 have received TB diagnoses or preventative treatment (6).

Afghanistan is a low-income country with an estimated population of 37 m people. There is a disproportionately high burden of tuberculosis that greatly impacts peoples' lives, with annually ~13,000 tuberculosis-associated deaths (7) and an estimated 9,800 deaths in 2020 (8). Countries with a high burden of TB and cases include Indonesia, China, Philippines, Pakistan, Nigeria and South Africa (9). The high burdens are associated with multiple factors, including cultural beliefs, illiteracy, low outreach and access to health facilities, stigma, and poverty (10). It has also been identified that TB infects women more than men in Afghanistan (10). This is similar to what's reported in other parts of the world. For instance, in a study conducted in China, a high rate of stigma among TB patients was observed, particularly among females (11). Similarly, in Peru, where TB is a high burdened disease, there is a considerable lack of knowledge among participants on the symptoms; this further emphasizes the need for health literacy and education awareness (12).

The response to the COVID-19 pandemic in Afghanistan has seen disruption in health services. For example, the polio program was suspended amidst the pandemic, later attributed to a spike in polio cases among children (13). Now, with the multiple waves of the pandemic, the crumbling healthcare system is struggling with a set of challenges, such as the global emergence of new variants of the novel coronavirus, oxygen and bed shortages (14).

Concurrently, the Taliban is dominating the country and causing significant political tension. Therefore, there is a lack of support and funding from international organizations because of the new government's dominance. Many health facilities are not properly functioning (15). Without external support, TB would remain a serious health issue. Therefore, the main objective of this study was to assess the Hospital's patients' knowledge, attitude, and practice toward TB in Kabul, Afghanistan, to report an updated overview of the TB burden in the country to the local and international communities and stakeholders. In addition, it provides an insight for the researchers to study the subject more in-depth.

## Methods

### Study Setting and Design

A cross-sectional descriptive study was conducted on Kabul residents who visited the adult outpatient departments (OPD) of public health facilities for various health services; this included, but was not restricted to, attendance for follow-up on a confirmed case of tuberculosis. Data collection took place from 1st January to 20th March 2022 from public health facilities such as (Jamhoriat, Ibni Sina, Maiwand, Estiqlal, Barchi, Ali Abad, Aryana, Amiri, Royal, Global, Shaikh Zayid, Wazir Akbar Khan, Indragandi, Bed Khair Khana, Sama, Khatumul Nabeyeen, Milat, Malalai, and Rabeya Balkhi).

### Selection Criteria

For this study, inclusion and exclusion criteria were all patients 18 years of age or above and were Outpatient departments referral. Exclusion criteria were patients under 18 and were not Outpatient departments referral.

### Data Collection

The survey questionnaire was adapted from a similar study conducted in Ethiopia (16). The questionnaire was modified slightly according to the context. Here, questions were asked in person, and data were recorded on paper forms (Supplementary File 1).

#### Sociodemographic Questions

The study asked basic sociodemographic questions such as age, gender, marital status, educational status, occupation, and average monthly income (afghani), which were categorized into three classes, namely, <1,000 (afghani), 1,000–3,000 (afghani), and >3,000 (afghani). The median monthly income in Kabul is 5,000 Afghani, equating to ~57.14 US dollars.

#### Knowledge Questions

We ask about TB mode of transmission, TB preventability, and TB symptoms. The knowledge grade was calculated by giving a (correct answer a score of 1 and a wrong answer a score of 0). We had nine points, so the maximum score was nine, and the minimum score was zero (0-9). We categorized the knowledge as follows—Poor knowledge (0–5) and Good knowledge (6–9). This scoring system was based on a similar study done in Ethiopia. The area under the receiver operator characteristic (ROC) curve (AUC) results of the knowledge were considered good and equalled 0.86.

#### Attitude Questions

We ask whether participants considered TB a dangerous disease for the community, whether TB transmission from human to human and the presence of stigma for TB patients. The attitude grade was calculated according to (true answer = one and wrong answer = 0). We had three points, so the maximum score was three, and the minimum score was zero (0–3). We categorized the attitude as follows [Positive attitude (2–3) and Negative attitude (0–1)]. The area under the ROC curve (AUC) attitude results were considered good and equalled 0.801.

#### Practice Questions

We ask about participant knowledge around good practices for controlling TB, including opening house windows regularly, opening car window's during traveling, TB screening, having TB health education, having TB, and action after having TB. The practice grade was calculated according to (true answer = one and wrong answer = 0). We had eight points, so the maximum score was eight, and the minimum score was zero (0–8). We categorized the practice as follows [Poor practice (0–4) and Good practice (5–8)]. The area under the practice's ROC curve (AUC) results were considered excellent and equalled 0.998.

### Sample Size and Sampling Type

All participants fulfilling the inclusion criteria were invited to participate. The sample size was calculated using Epi-Info. Kabul's population has an estimated 4 million 400 thousand with a 95% confidence level, 50% expected frequency, 1.0 effected size, and 5% margin of error. The measured required minimum sample size was 385 participants. The sampling method is chosen the non-probability convenient sampling for data gathering.

### Pilot Study

The questionnaire was pre-tested on a small sample of participants (70 participants) to confirm the practicability and clarity of questions. Expert researchers reviewed the questionnaire for content validity and considered their comments. For reliability, Cronbach's alpha was 0.753 for every questionnaire section, indicating an acceptable level of consistency. Observations of the pilot study were not included in the final analysis.

### Ethical Approval

Ethical approval was obtained from the Microbiology Department of Kabul University of Medical Sciences Research Committee (KUMS-MD-2241). Informed consent was taken from the participants before they participated in the study.

### Statistical Analysis

The knowledge scoring was based on the three knowledge questions. The extracted data was presented in an excel sheet, and data were analyzed using SPSS version 28. The optimal cutoff point was evaluated using the ROC curve. The area under the ROC curve (AUC) results were considered excellent for AUC values between 0.9 and 1, good for AUC values between 0.8 and 0.9, fair for AUC values between 0.7 and 0.8, poor for AUC values between 0.6 and 0.7 and failed for AUC values between 0.5 and 0.6 (17). Normality was tested using the Kolmogorov-Smirnov test, and histogram. For continuous non-normally distributed data, median and interquartile range (IQR) was used, and comparisons were made using the Mann-Whitney test. Results were summarized using counts (*n*) and percentages (%) for categorical data with comparisons made using the Chi-square or Fisher extract test. A value of *p* < 0.05 was considered statistically significant. The association between knowledge, attitude, and practices toward tuberculosis was tested using spearman correlations. The binary logistic regression model was interpreted using odds ratio (OR) and 95% confidence interval (CI).

## Results

### Baseline Characters of Participants

Of the 829 participants, 450 (54.3%) were males, 379 (45.7) were females, and the median age was 28 years (IQR = 20) and ranged from 9 to 80. The majority of our sample was unemployed participants (75.5%), but 54% of the participants had a monthly income >3,000 Afghanis (34.29$) Table 1.

**Table 1:** Baseline characters of participants (*N* = *829*).

**Variables**		**Total**	
		***N***	***%***
Age	Median; IQR (range)	28	20 (9–80)
Gender	Male	450	54.3
	Female	379	45.7
Marital status	Single	279	33.7
	Married	525	63.3
	Widow	25	3
Educational status	Can read and write	499	60.2
	Cannot read and write	330	39.8
Occupation	Employed	203	24.5
	Unemployed	626	75.5
Monthly income categories	<1,000	1	0.1
	1,000–3,000	139	16.8
	>3,000	448	54.0
Monthly income	Median; IQR (range)	6,000	6,000 (300–31,000)

*TB, Tuberculosis; IQR, interquartile range; N, Number*.

### Knowledge, Attitude, and Practices Toward Tuberculosis Among Hospital Outpatients

Overall, 727 (87.7%) participants had good TB knowledge, and 800 (96.5%) participants had a positive attitude toward TB treatment and control. Moreover, only 2 participants reported poor practices regarding TB prevention. The baseline characters are shown in Table 2.

**Table 2:** Knowledge, attitude, and practices toward tuberculosis among hospital outpatients in Kabul, Afghanistan (*N*= *829*).

**Variables**		**Total**	
		***N***	***%***
**1.Knowledge**			
What is the TB mode of transmission? Inhalation droplets	No	32	3.9
	Yes	797	96.1
What is the TB mode of transmission? Heredity	Yes	519	62.6
	No	310	37.4
What is the TB mode of transmission? Shaking someone's hand	Yes	398	48
	No	431	52
What is the TB mode of transmission? Sharing the food/drink	Yes	727	87.7
	No	102	12.3
Is TB preventable?	No	16	1.9
	Yes	813	98.1
What are the main symptoms of TB? Cough that lasts more than 3 weeks	No	28	3.4
	Yes	801	96.6
What are the main symptoms of TB? Fever	No	117	14.1
	Yes	712	85.9
What are the main symptoms of TB? Loss of appetite	No	145	17.5
	Yes	684	82.5
What are the main symptoms of TB? Night sweats	No	196	23.6
	Yes	633	76.4
Knowledge score	Median; IQR (range)	6.5	1 (1–9)
Knowledge grading	Poor knowledge	102	12.3
	Good knowledge	727	87.7
**2.Attitude**			
Is TB a dangerous disease for the community?	No	33	4.0
	Yes	796	96.0
Does TB transmit from human to human?	No	17	2.1
	Yes	812	97.9
Should TB patients get stigmatized?	Yes	784	94.6
	No	45	5.4
Attitude score	Median; IQR (range)	2	0 (0–3)
Attitude grading	Positive attitude	800	96.5
	Negative attitude	29	3.5
**3.Practice**			
Does your house have a window?	No	5	0.6
	Yes	824	99.4
Do you open your home window regularly?	No	3	0.4
	Yes	826	99.6
Do you open car window's during traveling?	No	91	11
	Yes	738	89
Have you ever screened for TB?	No	724	87.3
	Yes	105	12.7
Have you ever got health education about TB?	No	721	87
	Yes	108	13
If you have TB, what do you do?	Consult traditional healers	8	1
	Consult health worker	821	99
If you have TB, what measures would you do for the family and community?	Cover my mouth and nose during coughing and sneezing	829	100
Practice score	Median; IQR (range)	6	0 (4–8)
Practice grading	Poor Practice	2	0.2
	Good Practice	827	99.8

### The Extent of TB Knowledge and Knowledge Predictors

Males significantly had better knowledge more than females (*n* = 369 and 358, respectively) (Overall *P*-value = 0.001). Belonging to the “>3,000” Afghani monthly income category significantly had better knowledge more than other categories (1,000–3,000 and <1,000) (*n* = 403, 111, and 1) (Overall *P*-value = 0.006). A positive attitude was significantly associated with good knowledge (Overall *P*-value = 0.019).

Regarding the binary logistic regression, young age, being a male, belonging to the “1,000–3,000” Afghani monthly income category, and having a positive attitude were significant predictors of good TB knowledge (*P*-value = 0.009, 0.000, 0.003, and 0.009), respectively (Table 3).

**Table 3:** Knowledge and associated factors (*N* = *829*).

**Variables**		**Knowledge grading**					**Binary logistic regression**			
		**Poor knowledge**		**Good knowledge**		^*^Overall *p***-value**	***P*** **-value**	**Odds Ratio**	**95% confidence interval**	
		***N***	**%**	***N***	**%**				**Lower**	**Upper**
Age	Median; IQR (range)	26	27 (12–70)	28	18 (9–80)	0.604	0.009	0.964	0.938	0.991
Gender	Male	81	18	369	82	0.001	0.000	0.300	0.156	0.577
	Female	21	5.5	358	94.5		*Ref*			
Marital status	Single	41	14.7	238	85.3	0.178	0.677	0.054	8.445	0.677
	Married	60	11.4	465	88.6		1.969	0.194	19.947	1.969
	Widow	1	4	24	96		*Ref*			
Educational status	Can read and write	67	13.4	432	86.6	0.237	0.430	1.318	0.664	2.616
	Cannot read and write	35	10.6	295	89.4		*Ref*			
Occupation	Employed	25	12.3	178	87.7	1	0.581	1.177	0.660	2.100
	Unemployed	77	12.3	549	87.7		*Ref*			
Mentioned the monthly income	No	29	12	212	88	1	0.879	1.036	0.655	1.640
	Yes	73	12.4	515	87.6		*Ref*			
Monthly income categories	<1,000	0	0	1	100	0.006	0.003	2.171	1.306	3.608
	1,000–3,000	28	20.1	111	79.9					
	>3,000	45	10	403	90					
Monthly income	Median; IQR (range)	5,000	5,500 (1,000–20,000)	6,000	6,000 (300–31,000)	0.039	0.763	1.000	1.000	1.000
Attitude score	Median; IQR (range)	2	0 (1–3)	2	0 (0–3)	0.298	0.161	0.498	0.187	1.321
Attitude grading	Positive attitude	94	11.8	706	88.3	0.019	0.009	6.035	1.572	23.167
	Negative attitude	8	27.6	21	72.4		*Ref*			
Practice score	Median; IQR (range)	6	0 (5–8)	6	0 (4–8)	0.494	0.580	1.092	0.800	1.492
Practice grading	Poor practice	0	0	2	100	1	N/A			
	Good practice	102	12.3	725	87.7					

*TB, tuberculosis; IQR, interquartile range; N, Number.*

* *Overall P-value of Mann-Whitney test and Chi-square or Fisher extract test*.

Belonging to a can read and write education level is predicted to have good knowledge 1.318 times more than belonging to a cannot read and write level (95% CI: 0.664–2.616). Being employed was predicted to have good knowledge 1.177 times more than being unemployed (95% CI: 0.660–2.100). Belonging to the “ <1,000” Afghani monthly income category expected to have good knowledge 2.171 times more than other categories (1,000–3,000 and >3,000) (95% CI: 1.306–3.608). A positive attitude was expected to have good knowledge 6.035 times more than a negative attitude (95% CI: 1.572–23.167) (Table 3).

### Attitude Toward TB and Attitude Predictors

Males significantly had positive attitude more than females (*n* = 427 and 373, respectively) (Overall *P*-value = 0.007). Good knowledge was significantly associated with a positive attitude (Overall *P*-value = 0.019) (Table 4).

**Table 4:** Attitude and associated factors (*N*= *829*).

**Variables**		**Attitude grading**					**Binary logistic regression**			
		**Positive attitude**		**Negative attitude**		^*^Overall ***P*****-value**	***P*** **-value**	**Odds ratio**	**95% confidence interval**	
		***N***	**%**	***N***	**%**				**Lower**	**Upper**
Age	Median; IQR (range)	28	20 (9–80)	23	10 (13–70)	0.598	0.716	0.991	0.943	1.041
Gender	Male	427	94.9	23	5.1	0.007	0.018	4.726	1.310	17.043
	Female	373	98.4	6	1.6		*Ref*			
Marital status	Single	270	96.8	9	3.2	0.572	0.762	0.909	0.491	1.684
	Married	505	96.2	20	3.8					
	Widow	25	100	0	0					
Educational status	Can read and write	478	95.8	21	4.2	0.183	0.027	10.761	1.317	87.953
	Cannot read and write	322	97.6	8	2.4		*Ref*			
Occupation	Employed	198	97.5	5	2.5	0.509	0.024	0.250	0.075	0.831
	Unemployed	602	96.2	24	3.8		*Ref*			
Mentioned the monthly income	No	232	96.3	9	3.7	0.836	0.813	1.102	0.494	2.455
	Yes	568	96.6	20	3.4		*Ref*			
Monthly income categories	<1,000	1	100	0	0	0.909	0.675	1.267	0.420	3.822
	1,000–3,000	135	97.1	4	2.9					
	>3,000	432	96.4	16	3.6					
Monthly income	Median; IQR (range)	6,000	6,000 (300–31,000)	5,000	5,250 (3,000–20,000)	0.905	0.675	1.000	1.000	1.000
Knowledge score	Median; IQR (range)	7	1 (3–9)	6	2 (1–8)	0.107	0.804	0.940	0.576	1.533
Knowledge grading	Poor knowledge	94	92.2	8	7.8	0.019	0.193	2.493	0.631	9.852
	Good knowledge	706	97.1	21	2.9		*Ref*			
Practice score	Median; IQR (range)	6	0 (4–8)	6	1 (5–8)	0.021	0.054	0.530	0.277	1.012
Practice grading	Poor practice	2	100	0	0	1	N/A			
	Good practice	798	96.5	29	3.5					

*TB, tuberculosis; IQR, interquartile range; N, number*.

* *Overall P-value of Mann-Whitney test and Chi-square or Fisher extract test*.

Regarding the binary logistic regression, being a male, belonging to a can read and write education level, and being employed were significant predictors of positive TB attitude (*P*-value = 0.018, 0.027, and 0.024), respectively (Table 4). Being a male was predicted to have a positive attitude 4.726 times more than being a female (95% CI: 1.310–17.043). Belonging to a can-read and write education level was predicted to have a positive attitude 10.761 times more than belonging to a cannot read and write level (95% CI: 1.317–87.953) (Table 4).

### Practice Toward TB and Practice Predictors

All single participants showed good practices against TB 279 (100%). Males had non-significant good practice more than females (Overall *P*-value = 1). Overall, very good practices against the prevention of TB were noted throughout (Table 5).

**Table 5:** Practice and associated factors (*N*= *829*).

**Variables**		**Practice grading**					**Binary logistic regression**			
		**Poor practice**		**Good practice**		^*^Overall *p*-value	***P*** **-value**	**Odds ratio**	**95% confidence interval**	
		***N***	**%**	***N***	**%**				**Lower**	**Upper**
Age	Median; IQR (range)	N/A		28	20 (9–80)	0.929	0.809	1.013	0.911	1.128
Gender	Male	1	0.2	449	99.8	1	0.903	1.188	0.074	19.055
	Female	1	0.3	378	99.7		*Ref*			
Marital status	Single	0	0	279	100	0.56	0.523	0.563	0.096	3.289
	Married	2	0.4	523	99.6					
	Widow	0	0	25	100					
Educational status	Can read and write	0	0	499	100	0.158	N/A			
	Cannot read and write	2	0.6	328	99.4					
Occupation	Employed	1	0.5	202	99.5	0.430	0.425	3.094	0.193	49.691
	Unemployed	1	0.2	625	99.8		*Ref*			
Mentioned the monthly income	No	1	0.4	240	99.6	0.497	0.528	2.446	0.152	39.262
	Yes	1	0.2	587	99.8		*Ref*			
Monthly income categories	<1,000	0	0	1	100	0.855	N/A			
	1,000–3,000	0	0	139	100					
	>3,000	1	0.2	447	99.8		*Ref*			
Monthly income	Median; IQR (range)	N/A		6,000	6,000 (300–31,000)	0.285	0.870	0.962	0.603	1.533
Knowledge score	Median; IQR (range)	N/A		7	1 (1–9)	0.407	0.610	1.348	0.429	4.237
Knowledge grading	Poor knowledge	0	0	102	100	1	N/A			
	Good knowledge	2	0.3	725	99.7					
Attitude score	Median; IQR (range)	N/A		2	0 (5–8)	0.005	0.015	0.031	0.002	0.506
Attitude grading	Positive attitude	2	0.3	798	99.8	1	N/A			
	Negative attitude	0	0	29	100					

*TB, tuberculosis; IQR, interquartile range; N, number*.

* *Overall P-value of Mann-Whitney test and Chi-square or Fisher extract test*.

Regarding the binary logistic regression, attitude score was found to be a significant predictor related to the practices toward TB [OR = 0.031 (95% CI: 0.002–0.506), *P*-value = 0.015] as shown in Table 5.

Young participants were expected to have a good practice 1.013 times more than old participants (95% CI: 0.911–1.128). A male was predicted to have a good practice 1.188 times more than a female (95% CI: 0.074–19.055). Being employed was predicted to have a good practice 3.094 times more than being unemployed (95% CI: 0.193–49.691) (Table 5).

### Correlations Between Knowledge, Attitude, and Practices Toward Tuberculosis

A strong positive correlation was noted between knowledge grading and knowledge score [Rho (R)-value = 0.696 and *P*-value = 0.001]. A low positive correlation was noted between practice grading and practice score (*R*-value = 0.152 and *P*-value = 0.001). A strong negative correlation was noted between attitude grading and attitude score (*R*-value = −0.747 and *P*-value = 0.001) (Table 6).

**Table 6:** Spearman correlations between knowledge, attitude, and practices toward tuberculosis.

		**Knowledge grading**	**Attitude grading**	**Practice grading**	**Knowledge score**	**Attitude score**	**Practice score**
Knowledge grading	Correlation coefficient	1					
	*P*-value						
Attitude grading	Correlation coefficient	−0.089	1				
	*P*-value	0.011					
Practice grading	Correlation coefficient	−0.018	0.009	1			
	*P*-value	0.596	0.788				
Knowledge score	Pearson correlation	0.696	−0.068	0.018	1		
	*P*-value	0.001	0.050	0.614			
Attitude score	Correlation coefficient	0.033	−0.747	−0.095	0.039	1	
	*P*-value	0.337	0.001	0.006	0.261		
Practice score	Correlation coefficient	0.016	−0.064	0.152	0.051	0.024	1
	*P*-value	0.641	0.065	0.001	0.141	0.484	

## Discussion

In this study, most participants had good knowledge (87.7%) and attitude (96.5%) toward TB. Similarly, participants ranked highly overall for TB practice. Higher monthly income and being female were observed predictors for higher scores.

However, this may not genuinely reflect the extent of TB KAP among the participants, as the country is going through unique circumstances. The Taliban regime has come into power. The dominance was followed by sanctions from different countries and international organizations, which resulted in funding suspension. Since then, the already-fragile healthcare system has not been functioning properly. The country's largest healthcare services provider “Sehatmandi” program, has lost its full functionality (14). The program aimed to provide essential health services to hospitals nationwide. Considering the unfolding events in Afghanistan and the lack of healthcare functionality, TB patients may remain undiagnosed. There is little or no health promotion activity to raise awareness and provide useful information about TB. Moreover, TB may spread among internally displaced refugees who are displaced because of conflict and war; thus, it may further wreak havoc on the healthcare system.

The finding of this study regarding knowledge about TB is reported higher than in a study done in the Mecha district of Ethiopia (also a low-income country), where 54% of participants had good knowledge about TB (16). However, the present finding is in line with another Ethiopian study carried out in the Shinile town of eastern Ethiopia. The majority of the participants had good knowledge about TB (18). A further cross-sectional study in Southern Ethiopia showed that 48.6% of the participants were considered to have good knowledge of TB (19). In this latter study, the score was significantly associated with gender and age. Other differences could be due to socio-economic, cultural, and environmental factors, particularly observed in a study conducted in the Gambia, where two-thirds of all participants had good or appropriate knowledge. Good TB knowledge was associated with age, marital status, education, employment, and a good TB attitude and practice (20).

Regarding the attitude, 96.5% of the participants had a good attitude toward TB. Females had a better attitude than their male counterparts; this is in line with a study conducted in Shingle town, eastern Ethiopia, where half of the participants considered TB a very serious disease (18). In another study conducted in the North Mecha district of Ethiopia, 68% of the participants stated a good attitude, and 44% were ranked as having good practice (16). Similarly, in a study conducted in Thailand, 47.9% of study participants were categorized with a high level of attitude TB (21).

In this Afghanistan study, those participants with a better attitude had good practice toward TB. Only a few participants reported poor practice regarding TB. Furthermore, male participants had better practice, but it was not statistically significant. Observed differences may reflect social dynamics, environment, and perceived access to healthcare facilities. These patients were interviewed in the health settings of Kabul, the capital of Afghanistan, where access to TB medication is available. However, this does not apply to all country contexts, especially rural ones.

In this study, 98% of the participants stated that TB is preventable in our study. This high number could be attributable to Kabul's awareness program and widely available treatment centers. The finding is similar to what is reported in Shinali town, where 79.3% of the participants responded that transmission of TB would be preventable (16). However, in another study conducted in Tepi General Hospital of Ethiopia, the majority (70%) of participants responded that its transmission is not preventable, while 92 (22.4%) replayed that covering the mouth and nose while coughing or sneezing is a possible method to prevent the transmissions (22).

Most participants (96.6%) stated cough as the main symptom, followed by fever and loss of appetite. Similar symptoms were reported by other participants in other studies (16, 20, 23).

Gender and level of education were associated with better knowledge of TB; this is in line with a study conducted in Khyber Pakhtunkhwa, Pakistan, where females and education level was correlated with better knowledge of TB (24).

Our study has several limitations. The study was conducted only in the capital, Kabul. The findings cannot be generalized to the whole country. Further studies are needed to provide a more in-depth assessment of TB in the country. Moreover, the study was done in hospital settings. Hence, there could be differences among participants in the community. Thus, another study could provide greater insight into this.

## Conclusions

The study concluded that participants had good knowledge, attitude, and practice toward TB. A recommendation for policymakers is that TB knowledge is high in this study population, which can be used as a stepping-stone for future health promotion activities. Future awareness-raising campaigns will need to be tailored to the needs and socio-economic circumstances of the target communities. This study can contribute to the evidence base that informs those campaigns. Further research is warranted to provide additional data from other Afghanistan populations to allow comparisons with the findings from this study; this should include vulnerable individuals who find it difficult to access healthcare, residents in rural areas where healthcare is especially limited, and internally displaced or refugee Afghan populations. These are all groups at higher risk of both tuberculosis and drug-resistant tuberculosis.

## Data Availability Statement

The original contributions presented in the study are included in the article/Supplementary Material, further inquiries can be directed to the corresponding author/s.

## Ethics Statement

The studies involving human participants were reviewed and approved by the Microbiology Department of Kabul University of Medical Sciences Research Committee (KUMS-MD-2241). The patients/participants provided their written informed consent to participate in this study.

## Author Contributions

ME and AN conceived the concept of the paper. ME, SA, and MK wrote the first draft and performed the analysis. KR, RN, and TD contributed data collection. AN and MH edited the second draft. All authors read and approved the final draft.

## Conflict of Interest

The authors declare that the research was conducted in the absence of any commercial or financial relationships that could be construed as a potential conflict of interest.

## Publisher's Note

All claims expressed in this article are solely those of the authors and do not necessarily represent those of their affiliated organizations, or those of the publisher, the editors and the reviewers. Any product that may be evaluated in this article, or claim that may be made by its manufacturer, is not guaranteed or endorsed by the publisher.
