# Supplementary material for: Knowledge, Attitude, and Practices Toward Tuberculosis Among Hospital Outpatients in Kabul, Afghanistan
Source: Front Public Health. 2022 Jul 11;10:933005. doi: 10.3389/fpubh.2022.933005 (PMC9309493; doi:10.3389/fpubh.2022.933005)
Supplement: Supplementary file 1 [file Data_Sheet_1.PDF]

Knowledge, attitude, and practice towards tuberculosis among patients visiting public health facilities in Kabul, Afghanistan

Tuberculosis (TB) is a major public health disease in Afghanistan. Almost 13 000 people die each year because of TB. Therefore, we aimed to conduct a survey among patients visiting public health facilities in Kabul to understand their (KAP) towards TB. The findings of this study would fill a huge gap of TB in Afghanistan. It will also help policymakers to make informed decisions about TB management in the country.

For more queries, feel free to contact:
Mohammad Yasir Essar
Yasir.essar@gmail.com

* Required

1. Do you want to participate in this survey? *

Mark only one oval.

- ( ) Yes
- ( ) No

Sociodemographic of the participants

2. Age *

____

3. Marital status *

Mark only one oval.

- ( ) Single
- ( ) Married
- ( ) Divorced
- ( ) Widow

4. Educational status *

Mark only one oval.

- Can read and write
- Cannot read and write

5. Occupation *

Check all that apply.

- Employed
- Unemployed

6. Monthly Income (Afghani) *

Knowledge of TB among participants

7. What is the TB mode of transmission? *

Check all that apply.

	Yes	No
Inhalation/droplets		
Heredity		
Shaking someone's hand		
Sharing the food/drink		

8. Is TB preventable? *

Mark only one oval.

- Yes
- No

9. What are the main symptoms of TB? *

Mark only one oval per row.

	Yes	No
Cough that last more than three weeks	⬭	⬭
Fever	⬭	⬭
Loss of appetite	⬭	⬭
Night sweats	⬭	⬭

Attitude of participants towards TB

Attitude of participants towards TB

10. Is TB a dangerous disease for the community *

Mark only one oval.

⬭ Yes

⬭ No

11. Is TB transmit from human to human? *

Mark only one oval.

⬭ Yes

⬭ No

12. Should TB patients get stigmatized? *

Mark only one oval.

⬭ Yes

⬭ No

Practice towards prevention of TB

Practice towards prevention of TB

13. Does your house has window? *

Mark only one oval.

- Yes
- No

14. Do you open your home window regularly? *

Mark only one oval.

- Yes
- No

15. Do you open car window’s during traveling? *

Mark only one oval.

- Yes
- No

16. Have you ever screened for TB? *

Mark only one oval.

- Yes
- No

17. Have you ever got health education about TB? *

Mark only one oval.

- Yes
- No

18. If you have TB, what do you do? *

Mark only one oval.

- Consult health worker
- Consult traditional healers
- Kept silent

19. If you have TB, what measures would you do for the family, community? *

Mark only one oval.

- Cover my mouth and nose during coughing and sneezing
- I will cough arbitrarily
- I do not know

This content is neither created nor endorsed by Google.

Google Forms
